# Exploring ethical, sustainable and effective foetal bovine serum alternatives for *in vitro* mammalian cell culture

**DOI:** 10.3389/ftox.2026.1776815

**Published:** 2026-03-10

**Authors:** Huiyuan Meng, Philip J. R. Day

**Affiliations:** 1 Division of Evolution, Infection and Genomics, Faculty of Biology, Medicine and Health, The University of Manchester, Manchester, United Kingdom; 2 Manchester Institute of Biotechnology, The University of Manchester, Manchester, United Kingdom; 3 Department of Medicine, University of Cape Town, Cape Town, South Africa

**Keywords:** cell culture, foetal bovine serum (FBS), human-derived alternatives, plant-based alternatives, regulatory challenges, serum-free media

## Abstract

Foetal bovine serum (FBS) has been widely used as a nutrient-rich supplement in mammalian cell culture for over 6 decades; however, its usage has increasingly raised various concerns and challenges related to quality variations, unethical collection practices, supply-demand imbalance and regulatory challenges. In recent years, alternatives have been investigated to reduce or replace FBS in mammalian cell culture. Starting from a comprehensive analysis of components of FBS and their functions in cell growth, this review compares the main types of FBS alternatives, i.e., human and animal-derived, plant-based alternatives and serum free media. Future perspectives discussed include the development of application-specific FBS alternatives, improvements in the quality and specialized formulation of FBS, optimization of existing alternatives and the establishment of databases and incentive mechanisms to facilitate the transition away from FBS. Lastly, the guidance for selecting appropriate FBS alternatives is also discussed.

## 1 *In vitro* cell culture

The development of *in vitro* cell culture techniques in the early 20th century revolutionized life sciences by enabling scientists to study cellular behaviour, physiology, and responses in a reproducible and manipulable environment (Subbiahanadar Chelladurai et al., 2021), and this has provided indispensable tools for cancer research, drug screening, vaccine production, and regenerative medicine (Segeritz CP, 2017; Weiskirchen et al., 2023). *In vitro* cell culture enables experimentation and observation of living cells under defined conditions and deepens understanding of disease mechanisms and cellular responses to therapeutic interventions (Geraghty et al., 2014; Fallahi et al., 2022). Using human and animal cell lines to screen the cytotoxicity of new drugs has also greatly decreased the time and expense required, offering a more efficient alternative to traditional *in vivo* testing (Fallahi et al., 2022). The cultivation of mammalian primary cells and immortalized cell lines marks a cornerstone of biomedical research (Yao and Asayama, 2016).

The development of mammalian cell culture technique has been paralleled with the evolution of cell culture media (Yao and Asayama, 2016). Maintaining mammalian cells *ex vivo* requires a precisely controlled physiological environment, including temperatures of 28 °C–39 °C, a pH of 7.2–7.4, and particularly a nutrient-rich media that can support cellular homeostasis (Weber et al., 2025; Bonnet et al., 2020). While 37 °C is standard for most mammalian cell lines, alternative temperatures are used for specific cell types, such as culturing COS-1 cells at 28 °C to enhance recombinant protein production, or porcine macrophages at 39 °C to approximate the higher body temperature of pigs (Fan et al., 2024; Natale and McCullough, 1998). Basal media provide essential nutrients, including carbohydrates (e.g., glucose, galactose), and nitrogen sources such as glutamine (Lee D. Y. et al., 2022). Early formulations, such as Tyrode’s and Locke’s solutions, provide only basic ionic balance and are insufficient for long-term cell maintenance (Men, 2024). Subsequent media, including Eagle’s Minimum Essential Medium (MEM), Dulbecco’s Modified Eagle Medium (DMEM), and Roswell Park Memorial Institute (RPMI) 1640, incorporated amino acids, vitamins, and buffering systems to better support cellular metabolism (Wijerathna-Yapa et al., 2025). Despite these advances, mammalian cells require exogenous growth factors, hormones, and complex macromolecules for survival and proliferation (Dos Reis et al., 2022; Bonnet et al., 2020). Accordingly, serum supplements remain essential for most mammalian cell cultures, as serum provides growth factors, hormones, amino acids, vitamins, and proteins that are difficult to replicate synthetically (Men, 2024; Lee D. Y. et al., 2022). Among various serum tested, given its rich composition and broad compatibility with diverse mammalian cell lines, foetal bovine serum (FBS) used at 5%–20% (v/v) has emerged as the most extensively used supplement in cell culture media (Lee D. Y. et al., 2022).

### 1.1 Composition and function of FBS

FBS is derived from bovine foetuses collected during the slaughtering process of pregnant cows (Yao and Asayama, 2016; Subbiahanadar Chelladurai et al., 2021; Lee et al., 2023). FBS comprises approximately 1,800 proteins and over 4,000 metabolites, reflecting its complex and nutrient-rich composition (Subbiahanadar Chelladurai et al., 2021). The composition of FBS is primarily influenced by maternal genetics, diet, and environmental factors (Liu et al., 2023; Brunner et al., 2010). Owing to its dynamic and undefined nature, researchers have focused on identifying the concentration of the key constituents and their functions—such as proteins, growth factors, hormones, amino acids, and vitamins—in modulating cellular homeostasis in culture. Table 1 presents the approximate average concentrations of each component and the likely variability across FBS lots.

**TABLE 1:** FBS components and their function.

Category	Compositions	Concentration per litre	Function (s)	References (s)
Serum proteins	Albumin	4,000–5,000 mg	Antioxidant protection	Lee D. Y. et al. (2022), Men (2024)
	Fibronectin	30 mg	Cell attachment, spreading	Parisi et al. (2020)
	Globulins (e.g., IgG)	500 mg	Pathogen neutralisation	Jazayeri et al. (2013)
	Kininogen	70–80 mg	Endothelial permeability and inflammatory signalling	Ponczek (2021)
	Haemoglobin	200–250 mg	Modulation of oxidative stress	Drvenica et al. (2022)
	α1-Antitrypsin (Protease Inhibitor)	3,500 mg	Reduction of protease-driven detachment or apoptosis	O'Brien et al. (2022)
	α2-Macroglobulin (Protease Inhibitor)	10 mg	Protease inhibition and preservation of matrix integrity	Vandooren and Itoh (2021)
Transport proteins	Transcortin	12 mg	Hormone and iron transport carrier, preventing oxidative stress	Subbiahanadar Chelladurai et al. (2021)
	Transferrin	20–220 mg	Ferric iron transportation	Stival et al. (2025)
	α1/ β1-Lipoprotein	19,000 mg	Lipid transportation	Liu et al. (2023), Rafnsdóttir et al. (2023)
Enzymes	ALT/GPT	5–10 IU	Converting alanine to pyruvate and glutamate. Cytotoxicity biomarker	Ozer et al. (2008)
	AST/GOT	40–50 IU	Transamination of aspartate to glutamate. Cytotoxicity biomarker	Chang et al. (2016)
	Lactate dehydrogenase	500–600 IU	Anaerobic glycolysis metabolism. Cytotoxicity biomarker	Allen et al. (1994)
Growth factors	EGF	0.2–2.0 µg	Cell growth, proliferation	Farooq et al. (2021), Boonstra et al. (1995), Storkebaum and Carmeliet (2004), Miell et al. (1997)
	FGF	10–40 µg	Cell growth, proliferation	Farooq et al. (2021)
	IGF	40–72 µg	Cell proliferation, inhibition of apoptosis	Miell et al. (1997)
	TGF	30–60 µg	Cell differentiation and extracellular matrix production	Miyazono et al. (1993)
Hormones	Steroid hormones (e.g., Corticosteroids, follicle-stimulating hormone, growth hormone, testosterone, thyroid)	Lot dependent	Regulating cell growth, protein synthesis and lipid metabolism	Pihl and Eker (1965), Jazayeri et al. (2013), Rosenblum and Carbone (1974)
	Insulin (bovine)	1–3 µg	Glucose uptake, lipid synthesis	Subbiahanadar Chelladurai et al. (2021)
Fatty acids and lipids	Cholesterol	300–800 mg	Crucial for membrane integrity and lipid metabolism	Lee D. Y. et al. (2022)
	Free and protein-bound fatty acids	Total 0.1–0.5 mmol	Membrane synthesis, energy production	Newsom et al. (2025)
	Triglycerides	300–930 mg	Energy storage	Newsom et al. (2025), Stival et al. (2025)
	Phospholipids	1,250–2,750 mg	Membrane synthesis	Newsom et al. (2025)
Carbohydrates	Fructose	11.1–33.0 mmol	Maintaining cellular homeostasis	Lee D. Y. et al. (2022)
	Glucose	1,000–2,000 mg		
	Mannose	50 µmol		
	Ribose	7 µmol		
Nitrogens	Amino acids	Lot dependent	Energy production	Qian and Saltzman (2004)
	Creatinine	20–30 mg		
	Purines	20–30 mg		
	Uric acid	20 mg		
Vitamins	Vitamin A	8.6 mg	Regulating cell differentiation *via* retinoic acid signalling	Liu et al. (2023)
	Vitamin C	0.04–0.1 mg	Collagen synthesis	
	Vitamin B group (e.g., Biotin, niacinamide. Nicotinic acid, folic acid, thiamine)	0.027–0.27 mg	Nucleotide synthesis and energy metabolism	
	Vitamin E	8.61 mg	Antioxidant	
Ions	Ca^2+^	1.5–4.0 mmol	Maintaining osmotic balance and homeostasis	Lee D. Y. et al. (2022), Mohamed et al. (2020)
	Cl^−^	100–110 mmol		
	K^+^	5–10 mmol		
	Na^+^	130–150 mmol		

These values represent approximate averages with a lot-to-lot variability in FBS.

ALT/GPT, alanine aminotransferase; AST/GOT, aspartate aminotransferase/glutamic oxaloacetic transaminase; EGF, epidermal growth factor; FBS, Foetal bovine serum; FGF, fibroblast growth factor; IGF, insulin-like growth factor; TGF, transforming growth factor.

#### 1.1.1 Serum proteins

All proteins exist in blood serum are known as serum proteins and are crucial for cell culture. as they provide essential carrier, protective, and regulatory functions (Liu et al., 2023; Lee D. Y. et al., 2022; Jazayeri et al., 2013; Parisi et al., 2020; Ponczek, 2021; Drvenica et al., 2022; O'Brien et al., 2022; Vandooren and Itoh, 2021). Bovine serum albumin (BSA), which constitutes the predominant protein in FBS at concentrations of 4,000–5,000 mg/L plays critical roles in cellular protection and nutrient transport (Wu et al., 2023; Xu et al., 2023; Lee D. Y. et al., 2022). BSA mitigates the cytotoxic effects of potentially harmful substance, including free fatty acids, heavy metals and toxins through high-affinity binding and sequestration (Wu et al., 2023). Moreover, it exhibits antioxidant activity by scavenging reactive oxygen species (ROS), thereby reducing oxidative stress (Xu et al., 2023). In addition, BSA serves as a carrier for essential molecules, such as fatty acids and peptides (Lee D. Y. et al., 2022). Fibronectin (30 mg/L) is an extracellular matrix glycoprotein that promotes cell attachment and spreading (Lee D. Y. et al., 2022). By presenting adhesive motifs and acting as a scaffold for growth-factor sequestration, fibronectin profoundly influences the morphology, survival signalling and differentiation programmes of adherent cells (Parisi et al., 2020). Immunoglobulins (IgG) (500 mg/L) minimize antibody-induced immune responses, making them suitable for culturing cells from diverse species (Liu et al., 2023; Jazayeri et al., 2013). Kininogen (70–80 mg/L) influences endothelial permeability, inflammatory signalling, protease activation cascades, and cell attachment (Ponczek, 2021). Haemoglobin in FBS (200–250 mg/L) functions as an oxygen carrier and thereby protects cells from oxidative stress (Drvenica et al., 2022). Both α1-antitrypsin (3,500 mg/L) and α2-macroglobulin (10 mg/L) are protease inhibitors that reduce protease-driven apoptosis, and preserve matrix integrity (Vandooren and Itoh, 2021; O'Brien et al., 2022).

#### 1.1.2 Transport proteins

Transport proteins in FBS facilitate the delivery of vital nutrients, minerals, hormones, and lipids to cultured cells (Liu et al., 2023; Lee D. Y. et al., 2022; Rafnsdóttir et al., 2023; Subbiahanadar Chelladurai et al., 2021). For instance, transcortin (12 mg/L) regulates the metabolism of steroid hormones, particularly cortisol and corticosterone (Subbiahanadar Chelladurai et al., 2021). Transferrin (20–220 mg/L) is an essential glycoprotein that plays a vital role in transporting ferric ions into haemoglobin (Subbiahanadar Chelladurai et al., 2021). α1-Lipoprotein is a high-density lipoprotein (HDL) that contributes to cholesterol homeostasis and mitigates oxidative stress (Liu et al., 2023). β1-Lipoprotein, also known as low-density lipoprotein (LDL), delivers essential lipids for cell proliferation and membrane synthesis (Lee D. Y. et al., 2022; Rafnsdóttir et al., 2023).

#### 1.1.3 Enzymes

Enzymes sustain metabolic homeostasis, mediating nutrient interconversion, and serving as sensitive indicators of cell viability and cytotoxicity, thereby influencing both the physiological relevance of growth media and the interpretability of *in vitro* experiments (Ozer et al., 2008; Chang et al., 2016; Allen et al., 1994). Alanine aminotransferase (ALT/GPT) (5–10 IU/L) activity catalyses the transamination between alanine and α-ketoglutarate for pyruvate and glutamate production, thus enabling energy generation under varying nutrient conditions (Ozer et al., 2008). Aspartate aminotransferase (AST/GOT) (40–50 IU/L) maintains redox balance by regulating the transamination of aspartate to oxaloacetate and glutamate (Chang et al., 2016). Lactate dehydrogenase (LDH) (500–600 IU/L) maintains lactate-pyruvate balance and redox homeostasis, especially under high-glycolytic conditions (Allen et al., 1994). ALT, AST and LDH act as cytotoxicity biomarkers of cellular injury and mitochondrial disfunction (Ozer et al., 2008; Chang et al., 2016; Allen et al., 1994).

#### 1.1.4 Growth factors

FBS stimulates cell division and promotes cell cycle progression by offering various growth factors, such as epidermal growth factors (EGFs), fibroblast growth factors (FGFs), insulin-like growth factors (IGFs), transforming growth factors (TGFs) and platelet-derived growth factor (PDGF) (Boonstra et al., 1995). Non-cancer (normal) cells require multiple specific growth factors for survival and division since they depend strongly on external stimulatory signals from serum (Lee et al., 2023). In contrast, cancer cell growth requires basal nutrients (e.g., amino acids, glucose and buffering system) and fewer growth factors in FBS than normal cells, as cancer cells display overactive growth factor receptors, and foetal growth factors can confound the assessment of cell-intrinsic oncogenic pathway effects (Newsom et al., 2025). Albeit that, foetal growth factors may accelerate cell proliferation, and lower the costs associated with cell production or experimental procedures (Lee D. Y. et al., 2022). EGFs in FBS (0.2–2.0 μg/L) stimulate cell proliferation, migration and differentiation by activating the EGFR pathway (Subbiahanadar Chelladurai et al., 2021; Storkebaum and Carmeliet, 2004; Boonstra et al., 1995). For instance, EGFs induce differentiation of mesenchymal stem cells into osteoblasts, chondrocytes or adipocytes (Boonstra et al., 1995). FGFs (10–40 μg/L) constitute a family of heparin-binding proteins that regulate cell proliferation through the FGF-receptor-heparan sulphate complex (Farooq et al., 2021). FGF 2 (basic FGF) sustains stem cell pluripotency and endothelial cell viability (Farooq et al., 2021). FBS contains approximately 40–72 μg/L of IGFs that promotes cell proliferation and suppresses apoptosis through the PI3K/AKT and MAPK signalling pathways (Miell et al., 1997). TGF (30–60 μg/L) is a cytokine that plays a pivotal role in regulating cell differentiation and extracellular matrix production (Miyazono et al., 1993). Activation of TGF-β receptor I/II serine-threonine kinases initiates the canonical Smad2/3 signalling cascade and MAPK pathways, thereby integrating with other growth factor networks (Miyazono et al., 1993).

#### 1.1.5 Hormones

Cell growth, protein synthesis and lipid metabolism, with late-gestation foetuses generally yielding higher hormone levels are modulated from steroid and peptide hormones (Jazayeri et al., 2013; Miell et al., 1997; Rosenblum and Carbone, 1974). Corticosteroids, constituting a major fraction of hormones in FBS (5–30 μg/L), modulate immune responses and regulate cell proliferation (Jazayeri et al., 2013; Pihl and Eker, 1965). Growth hormone (GH; 0.5–3 μg/L) influences fibroblast proliferation, protein synthesis, and lipid metabolism through direct receptor activation and stimulation of IGF-1 signalling (Miell et al., 1997). Testosterone (0.05–0.20 μg/L) promotes proliferation in androgen-responsive tissues *via* crosstalk with EGF and IGF pathways (Rosenblum and Carbone, 1974). As noted previously, insulin (1–3 μg/L) facilitates glucose uptake, lipid synthesis, and amino acid transport through activation of the PI3K/AKT and MAPK signalling cascades (Subbiahanadar Chelladurai et al., 2021).

#### 1.1.6 Lipids and fatty acids

Lipids in FBS, including cholesterol, fatty acids, triglycerides and phospholipids, contribute to cell membrane synthesis, and serve as energy sources and precursors for signalling molecules that regulate cellular proliferation and differentiation (Lee D. Y. et al., 2022; Newsom et al., 2025). Cholesterol (300–800 mg/L) supports membrane integrity and the formation of steroid hormones and bile acids (Lee D. Y. et al., 2022). Fatty acids (0.1–0.5 mM) in FBS are mostly bound to bovine albumin for phospholipid synthesis and ATP production *via* the tricarboxylic acid (TCA) cycle (Newsom et al., 2025). Triglycerides (300–930 mg/L) and phospholipids (1,250–2,750 mg/L) support membrane lipid synthesis and glycerol homeostasis (Newsom et al., 2025). Collectively, these FBS-derived lipids help sustain both biosynthetic and energetic demands of cultured cells, particularly under conditions where glucose availability is limiting (Lee D. Y. et al., 2022; Newsom et al., 2025).

#### 1.1.7 Amino acids, vitamins, and ions

Amino acids are essential for nucleotide production and protein synthesis in culturing cell lines (Qian and Saltzman, 2004). FBS contains a broad spectrum of essential amino acids, while non-essential amino acids are often additionally supplemented in culture media to support optimal cell growth and metabolic function (Weiskirchen et al., 2023). Although present in trace concentrations, vitamins and ions in FBS exert significant effects on cellular proliferation and metabolic activity (Lee D. Y. et al., 2022). Vitamin C (0.6 µM), for instance, functions as an antioxidant and cofactor in collagen synthesis and epigenetic regulation. B-group vitamins (0.1–1.0 µM) serve as coenzymes in nucleotide synthesis (van der Valk et al., 2010). Vitamin E (20 µM) acts as a lipid-soluble antioxidant, protecting membranes from oxidative damage (Lee D. Y. et al., 2022). Retinoic acid (vitamin A) is an essential supplement in cell culture media for differentiation of various epithelial cell lines (van der Valk et al., 2010). Together, these vitamins support cell proliferation, differentiation, metabolic activity, and viability in culture. Ions, such as Na^+^, K^+^, Fe^2+^/Fe^3+^, Mg^2+^, Ca^2+^, Cl^−^, HPO_4_^2-^/H_2_PO_4_^−^, and HCO_3_^−^ in FBS help to regulate osmotic balance and homeostasis in culture media, reducing cellular damage from shifts in osmotic pressure and pH (Lee D. Y. et al., 2022).

### 1.2 Challenges of FBS usage in mammalian cell culture

**FIGURE 1:**
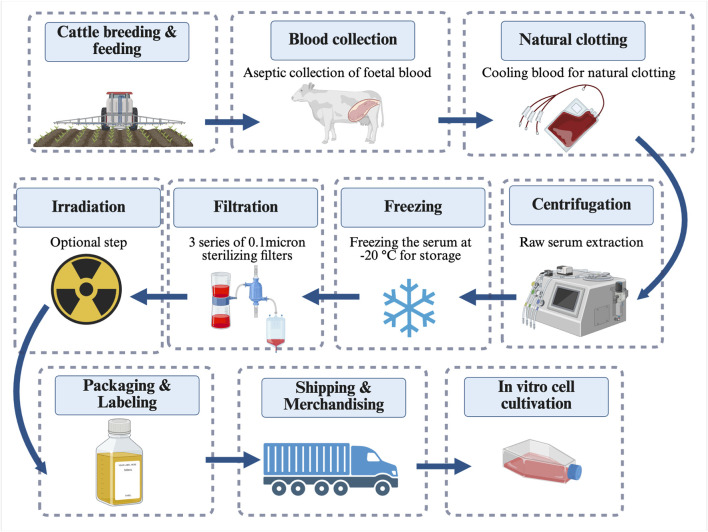
Diagram of FBS production process. FBS has been considered an effective growth factor supplement for *in vitro* cell and tissue culture media for 60 years. FBS is collected aseptically from the blood of bovine foetuses by cardiac puncture. Cooling and centrifugation are applied to separate the raw serum. For strict quality control, filtration is performed *via* three series of 0.1-micron membrane filters, as well as irradiation. Then, the FBS product is packaged, labelled, and transported. Created in BioRender (https://biorender.com/).

FBS is a meat-industry by-product obtained from foetuses of pregnant cows slaughtered for beef (van der Valk, 2022; Tancharoen et al., 2019; Versteegen et al., 2019). As illustrated in Figure 1, blood is aseptically collected *via* cardiac puncture from healthy foetuses following the slaughter of pregnant cows. The collected blood is cooled on ice to enable natural clotting and subsequently centrifuged to separate the raw serum. The raw serum is frozen at −20 °C, allowing manufacturers to store efficient batches of semi-processed material for subsequent processing. Filtration is performed using 0.1-micron membrane filters to obtain a high-quality product, and irradiation and heat inactivation are performed as additional quality control if necessary. FBS is then packaged, labelled, and shipped. Throughout the FBS manufacturing process, challenges related to quality nonconformities, health safety, ethical concerns and negative environmental impacts have collectively driven the implementation of regulation governing the use of FBS in cell culturing (Liu et al., 2023; Tancharoen et al., 2019; Subbiahanadar Chelladurai et al., 2021).

#### 1.2.1 FBS non-conformities and quality variation

Cellular heterogeneity in response to exogenous stimuli complicates the determination of whether observed differences in cell behaviour arise from the experimental variables under investigation or from unintended influences of the culture environment (Altschuler and Wu, 2010). Even subtle variations in culture media—such as nutrient composition, shear stress, or trace growth factors—can alter proliferation and differentiation kinetics, and introduce bias into experimental outcomes, thereby reducing reproducibility (Czapla et al., 2019). Therefore, rigorous control, and transparent reporting of media compositions are essential (Newsom et al., 2025; Czapla et al., 2019).

Significant compositional variabilities in proteins, amino acids and nutrients exists among commercial FBS products (Table 1) (Gstraunthaler et al., 2013; Gstraunthaler and Lindl, 2013; Liu et al., 2023; Zheng et al., 2006). Proteomic analyses have revealed about 2 to 4-fold differences between FBS from two manufacturers in the abundance of proteins associated with nutrient transport and cell protection, including apolipoprotein A-I, which mediates cholesterol transport; α-1-microglobulin, which is involved in detoxification; and α-1-antichymotrypsin, which contributes to antioxidant and anti-inflammatory properties (Liu et al., 2023). One study reported that transport protein transferrin levels in FBS ranged from approximately 1,800–2,200 mg/L across 13 lots sourced from the U.S.A. and Australia (Kakuta et al., 1997). In comparison, a recent analysis measured transferrin concentrations varying from 0 to 20 mg/L in 6 samples from the U.S.A. and Brazil, suggesting substantial variability closely associated with geographic origin and supplier (Stival et al., 2025). Manufacturer-dependent variation in growth factor composition further influences *in vitro* proliferation (Miell et al., 1997). For example, insulin-like growth factor binding protein (IGFBP) plays a central role in mediating IGF ligand–receptor interactions. IGFBP-4 was absent in two FBS lots that exhibited reduced growth-promoting capacity, but detectable in growth-promoting FBS lot from another supplier (Zheng et al., 2006). Another study found that VEGF concentrations in 6 FBS samples from the U.S.A. and Brazil varied between 6.43 and 20.85 μg/L, indicating more than a threefold difference (Stival et al., 2025). In addition, differences across eight FBS brands of South American, Australian, New Zealand origin in amino acid composition, particularly tyrosine, cysteine, and methionine have been shown to alter methylation patterns, mTOR signalling pathways, and baseline expression of inflammatory genes, such as *IL-8* (Liu et al., 2023). Systematic differences in total protein content, pH, and osmolality have also been reported among four FBS brands sourced from distinct geographic regions, including the U.S.A., Brazil, Australia and New Zealand (Lee D. Y. et al., 2022) (Table 2). This in turn modulates enzyme activity, ion balance and redox homeostasis in cultured cells, leading to distinct metabolic states (Gstraunthaler et al., 2013).

**TABLE 2:** Variation in the components of commercial FBS product.

Components	Sigma-Aldrich	Gibco	Biowest	Serana
Catalogue number	F6178	A5256801	S1300	S-FBS-AU-025
Origin	U.S.A.	Brazil	South Africa	Australia
Total protein (mg/mL)	36	40.6	40 ± 15	30.45
Endotoxin (EU/mL)	0.240	0.063	N/A	<10
pH	7.4	7.3	7.4 ± 0.6	6.8–8.2
Osmolality (mOsm/kg H_2_O)	308	308	322.5 ± 42.5	260–340

FBS, Foetal bovine serum.

#### 1.2.2 Health safety and ethical concerns

Safe handling is a challenge when using FBS in biomedical research, as animal-derived products can introduce pathogens and viruses into cell culture (Glotov et al., 2018; Brown, 2001). Despite advances in quality control, viral contamination in commercial FBS nowadays persists: for instance, Pestivirus genes were detected in 28 of 49 samples from 10 different suppliers (57%) (Kozasa et al., 2011), and in 2020, 84% of 124 tested FBS batches from 5 Argentinean brands contained Pestiviral RNA (Pecora et al., 2020). The risk of other contaminants, such as prions, bacteria, and exogenous extracellular vesicles, also exists. Human cells cultured in FBS may acquire a xenogeneic substance, potentially triggering immune responses in the patient that compromise the safety and efficacy of human stem cell transplantation (Martin et al., 2022). A recent outbreak of a pathogenic avian virus spreading among cows in several FBS-producing countries has been linked to severe human influenza cases with high mortality (PAHO/WHO, 2025; Martin et al., 2022; Paim et al., 2021). According to a PAHO/WHO report, between 2022 and 2024, 1,284 outbreaks of avian influenza A (H5N1) in mammals were reported across eight countries in the Americas, including Argentina, Brazil, Canada and the U.S.A. (PAHO/WHO, 2025). In 2024, the U.S.A. reported 67 human cases of H5N1, of which 40 were linked to exposure to diseased cattle (PAHO/WHO, 2025). Consequently, the usage of FBS sourced from these regions raises some concerns due to the potential risks of disease transmission *via* contaminated farm equipment and environmental exposure in cattle production systems (Eisfeld et al., 2024; Weber et al., 2025).

The killing and use of foetuses for commercial benefit raises serious ethical concerns (van der Valk, 2022). The industry claims the foetus is insensible due to oxygen deprivation when the mother’s circulation stops after slaughter (Gstraunthaler et al., 2013). However, scientific and ethical debates continue over whether the foetus may experience the distress during the serum extraction process (Gstraunthaler et al., 2013; FDA, 2006). Scientists, both from academia and animal welfare organisations, raise concerns to the neglect of animal welfare (Gstraunthaler et al., 2013; Prater, 2022; Weber et al., 2021).

#### 1.2.3 Supply issues and environmental sustainability

According to a recent market analysis report by Mordor Intelligence, global FBS market is valued at $2.35 billion in 2025 and is projected to reach $4.62 billion by 2030, representing a compound annual growth rate of 14.5%, though estimates vary across forecasting firms (Mordor Intelligence, 2025). Biopharmaceutical production, including cell culture manufacturing, accounted for the largest market segment (46.89%) in 2024 (Mordor Intelligence, 2025). FBS demand is expected to grow by 5%–7% annually, consistently exceeding supply due to constrained raw-material availability and market expansion, as estimates suggest an annual slaughter anywhere from one to more than two million foetal calves (Lee D. Y. et al., 2022; Jochems et al., 2002; Weber et al., 2025). Therefore, the imbalance between limited supply and rising demand continues to drive up retail prices (Brunner et al., 2010; M bioserviceS, 2015).

Most FBS suppliers are based in South Africa and Central America to leverage a well-developed cattle industry; however, meeting the high demand from laboratories in the U.S.A. (accounting for 37.2% of global sales) and rapidly expanding Asia-Pacific market incurs substantial transcontinental shipping costs (Brunner et al., 2010; Liu et al., 2023). In countries such as Korea, where livestock slaughter is prohibited during pregnancy, FBS must be entirely imported (Lee D. Y. et al., 2022).

FBS production, transportation and processing contribute to significant energy consumption, greenhouse gas emissions, and other negative environmental impacts (Lee et al., 2024; Lee et al., 2023). Cattle farming is a major source of methane emissions in agriculture (Lee D. Y. et al., 2022; Lee et al., 2023). Purifying FBS through labour-intensive manufacturing processes has been proposed as an alternative approach, but it inevitably increases costs (Cassotta et al., 2022). For instance, the price of premium-grade FBS with low endotoxin has risen to approximately $3,200 per 500 mL, five times higher than that of standard FBS (Mordor Intelligence, 2025).

### 1.3 Need for FBS alternatives

Regulatory frameworks and guidelines have been developed to address these aforementioned issues and promote the development of safer animal-derived products, such as the specific criteria for clinical-grade FBS established by the U.S.A. Food and Drug Administration (FDA) (Lee et al., 2023; Tannenbaum and Bennett, 2015; Poh and Stanslas, 2024), which complies with Good Manufacturing Practice (GMP) standards (DiNicolas, 2015; Schallmoser et al., 2020) and similar regulatory requirements are also enforced in the EU (EMA/CHMP/BWP, 2013). Many regulatory bodies, academic communities and funding agencies recommend reducing or replacing FBS with alternatives to foster more ethical research practices (Siegel, 2016). The National Centre for the Replacement, Refinement, and Reduction of Animals in Research (NC3Rs) in the UK, for instance, encourages researchers to reduce the use of animals or animal-derived products in scientific research (BIS/DH, 2014; Poh and Stanslas, 2024). According to Directive 2010/63/EU, the EU strongly recommends replacing animal-derived products with alternatives whenever possible (Louhimies, 2010).

## 2 FBS alternatives

Development of FBS alternatives can be traced back to the early 1980s–1990s, when researchers started exploring serum-free culture system and chemically defined media (Bjare, 1992; Tateishi et al., 2008). Recently, a systematic framework has been proposed for the rational development of FBS alternatives (Subbiahanadar Chelladurai et al., 2021): (1) Characterizing and quantifying the key compositions of commercial FBS, such as proteins, growth and hormones. (2) Identifying nutrients required for cell growth, proliferation and attachment, and sourcing them from defined, ethical, and scalable origins. (3) Screening FBS substitutes to measure the compositional similarity to FBS. In line with the abovementioned framework, measurable progress has been made in the development of main FBS alternatives, i.e., animal-, human-derived, and plant-based alternatives, and chemically defined media (Subbiahanadar Chelladurai et al., 2021). The most typical example(s) in each category and their costs, cell line specificity, ethical consideration, advantages and limitations are shown in Table 3.

**TABLE 3:** Comparison of FBS alternatives with FBS.

Parameters	Animal-derived alternatives		Human-derived alternatives	Plant-based alternatives	SFM
Typical example(s)	ABS	Earthworm HI-CF	hPL	*Aloe vera* extracts	CD media, PF media, ACF media, XF media.
Costs effective (compared with FBS price of Gibco™ $572/500 mL^a^)	Lower. Capricorn Scientific $95/500 mL	Higher.	Higher. Bioscience $1533/500 mL	Higher.	Varied. Invitrogen $197/500 mL HIMEDIA $1027/500 mL
Compatibility	Used in the cell culture when specific antibodies are required	Suitable for suspension cell culture.	Better compatibility for human cell (e.g., MSC) culture and clinical application.	Promoting regeneration of MSC. Successful for epithelial cell lines of LLC-PK1 and CaCo-2	Supporting various cell types, including neuronal lineages, fibroblasts, and cancer cells. Primary or stem cells require adaptation.
Ethical consideration	Byproduct of meat production. Concern raising only for those advocating reduction or elimination of animal-derived products.	Invertebrate-derived products may be regarded as more ethically acceptable than mammalian sources. But large-scale exploitation can still raise ecological and ethical questions.	Regulatory and ethical hurdles: sourcing and processing human blood products.	In accordance with ethical compliance and 3R principles.	In accordance with ethical compliance and 3R principles.
Components	Less growth factors compared with FBS. Similar batch variability.	No presence of immunoglobulin and fibronectin. Presence of vitamins, proteins, lipids, osmotic regulators and immune-protective molecules.	Higher IGF. Batch variability.	Abundant vitamins, amino acids, enzymes, polysaccharides and glycoproteins.	Well-defined components: high reproducibility. PF media applicable for hybridoma cells.
Health safety risk	Potential risk of zoonotic transmission.	Contamination of bacteria, fungi, or viruses.	Transmitting infectious diseases from human (e.g., HIV).	Limited cytotoxicity effects.	Enhanced consistency and reduced risk of contamination.
Limitations	Batch variation, low levels of growth factors (not applicable for MSC cells and human osteosarcoma cells.	Fibrinolytic enzymes disturb attachment and growth of adherent cell. Not for suspension cells.	Immunogenic residues.	Undefined composition in plant source and processing. Lack of standardization for clinical use. Applicability in various cell-line culturing is insufficiently understood.	PF media has no albumin or other protein, which slows cell growth. Cell adaptation challenges. Challenges in mimicking the FBS composition.
Reference(s)	Gstraunthaler (2003), Yu et al. (2013)	Cooper et al. (2002), Rossan Mathews et al. (2024)	Witzeneder et al. (2013), Shanbhag et al. (2020), Du et al. (2022), Schallmoser et al. (2020)	Georgiev et al., (2018), Badakhsh et al. (2014), Combe et al. (2024)	Gstraunthaler (2003)

ABS, Adult bovine serum; ACF, animal component-free; CD, chemically defined; FBS, Foetal bovine serum; HI-CF, heat inactivated coelomic fluid; hPL, human platelet lysate; IGF, insulin-like growth factor; MSC, mesenchymal stem cell; PF, protein-free; SFM, serum free media; XF, xeno-free.

^a^ Accessed at https://www.fishersci.com/shop/products/fetal-bovine-serum-value-one-shot-format/A5256701 on 15th December 2025.

### 2.1 Animal-derived alternatives

Animal-derived alternatives mainly include animal sera, platelet lysates and tissue extracts (Wijerathna-Yapa et al., 2025), supplying essential growth factors, hormones and proteins for cell proliferation and survival, and are more cost-effective than FBS. However, they may also pose contamination risks and exhibit variability in quality, which can compromise reproducibility, and sourcing from other animals still raises ethical concerns (Wijerathna-Yapa et al., 2025; Cooper et al., 2002; Yu et al., 2013).

Adult bovine serum (ABS), obtained from adult cattle, is a less controversial bovine serum supplement compared to other bovine-derived sera in cell culture (Cooper et al., 2002; Yu et al., 2013; Pakkanen, 1994). ABS contains higher levels of immunoglobulin (e.g., IgG, IgM, IgA) and overall antibody concentrations compared with FBS due to the mature immune system of adult cattle (Yu et al., 2013). Thus, it is suitable for studies requiring an immune-complex environment or cytokine responses, such as macrophage assays, and for stimulating adult mammalian plasma composition in immunotoxicology and vaccine response research (Gstraunthaler, 2003; Yu et al., 2013). At approximately one-fifth the cost of FBS, ABS is also cost-effective (Capricorn, 2026). However, ABS contains lower levels of growth factors (i.e., IGF: 10–30 μg/L, TGF: 15–25 μg/L), and this limits the proliferation of specific cell types, including MSCs and human osteosarcoma cells (Shehzadi et al., 2024; Pakkanen, 1994). Moreover, bovine serum derived from the supernatant of clotted blood still exhibits batch-to-batch variation and is obtained from animals that are subjected to potential suffering within the agricultural sector (Gstraunthaler, 2003).

Earthworm coelomic fluid is an emerging invertebrate-derived alternative containing various types of vitamins (riboflavin) that contributes to immune-protection (phagocytosis, encapsulation) and wound-healing research (Cooper et al., 2002). However, the exact concentration of components in earthworm coelomic fluid remain largely unavailable (BIRAC, 2025), and its fibrinolytic enzyme has been shown to impede cell attachment, limiting its applicability for culturing adherent cells (Rossan Mathews et al., 2024).

### 2.2 Human-derived alternatives

Human-derived alternatives provide ethically favourable supplements by eliminating animal-derived components (Golshan et al., 2025). Human serum (HS) and human platelet lysate (hPL), obtained from donor blood, preserve essential growth factors, such as PDGF (Golshan et al., 2025). Both HS and hPL reduce cell population doubling times of fibroblasts and maintain the differentiation potential of adipose tissue-derived stem cells (ASCs) compared to FBS (Witzeneder et al., 2013).

The HS is the aqueous portion of human blood deprived of clotting factors and is preferred for human cell cultures and studies that require a physiologically relevant environment for the cells (Psychogios et al., 2011). Table 4 compares the concentrations of protein, growth factors and hormones in bovine and human blood derived products, including FBS, ABS, human cord blood serum, HS and hPL. Human serum albumin (HSA) is the most abundant protein in HS that regulates osmotic pressure and transports a wide range of molecules (e.g., fatty acids, hormones) (Joh et al., 1986; Cavadas et al., 2011). Besides, HS contains higher IGF-1 levels (120–400 μg/L in adults), contributing to its stronger mitogenic potential compared to FBS in some types of cells (Tateishi et al., 2008). 15% HS promoted a 2.5-fold increase in mesenchymal stromal cells (MSCs) proliferation compared with the same concentration of FBS (Tateishi et al., 2008). HS has also been identified as more effective in terms of spheroid formation and cancer cell invasion (Mense and Rosol, 2018). Human cord blood serum has a high FGF-1 (500–700 μg/L) level to support rapid embryo growth (Farooq et al., 2021). In adults, however, HS usually has detectable but lower levels of FGFs (0.1–10.0 μg/L), EGFs (0.78 μg/L in males and 0.60 μg/L in females) and TGFs (1–5 μg/L) (Psychogios et al., 2011; Miyazono et al., 1993). Cortisol levels in adults (100–200 μg/L in the morning, 30–100 μg/L during the night) are higher than in the foetus (31.7 μg/L) (Rosenblum and Carbone, 1974). In addition, HS is nearly twice as expensive (Life Science Production, S-117A-US, £722.70) as FBS and less readily available (Heger et al., 2018).

**TABLE 4:** Concentrations of components in FBS, ABS, human cord blood serum, HS, and hPL.

Components	FBS	ABS	Human cord blood serum	HS	hPL	References(s)
Total protein	30–50 g/L	50–85 g/L	40–60 g/L	60–83 g/L	65–85 g/L	Cavadas et al. (2011), Yu et al. (2013)
Growth factors	EGF: 0.2–2.0 μg/L FGF: 10–200 μg/L IGF: 40–72 μg/L TGF: 30–60 μg/L	IGF: 10–30 μg/L TGF:15–25 μg/L	FGF-1: 500–700 μg/L IGF-1: 26–86 μg/L (21–34 weeks of gestation), 70–80 μg/L (before birth)	EGF: 0.78 μg/L (males) 0.60 μg/L (females) FGF: 0.1–10.0 μg/L IGF: 120–400 μg/L TGF: 1.5 μg/L	EGF: 0.07 μg/L FGF: 14 μg/L IGF: 125 μg/L	Storkebaum and Carmeliet (2004), Joh et al. (1986), Mohamed et al. (2020), Hsieh et al. (2009), Boonstra et al. (1995), Miell et al. (1997)
Hormones	Cortisol: 20 μg/L	Cortisol: 5–10 μg/L	Cortisol: 31.7 μg/L	Cortisol: 100 μg/L (morning), 30–100 μg/L (night)	Cortisol: 72.8–77.3 μg/L	Ferrara et al. (1992), Peters et al. (2022)
Ions	Na^+^: 130–150 mM K^+^: 5–10 mM	Na^+^:140 mM K^+^: 5 mM	Na^+^: 135–145 mM K^+^: 4–5 mM	Na^+^: 135–145 mM K^+^: 3.5–5.0 mM	Na^+^: 150 mM K^+^: 3.5–5.0 mM	Lee D. Y. et al. (2022), Mohamed et al. (2020)

ABS, adult bovine serum; EGF, epidermal growth factor; FBS, Foetal bovine serum; FGF, fibroblast growth factor; HS, human serum; hPL, human platelet lysate; IGF, insulin-like growth factor; TGF, transforming growth factor.

The hPL is collected from activated healthy human donor platelets with abundant growth factors, thereby minimizing the risks of xenogeneic immune responses, zoonotic transmission, and the ethical issues associated with animal-derived serum (Bieback, 2013). hPL promotes MSCs adhesion, survival, and proliferation, providing a safer and more clinically compatible alternative for therapeutic applications (Gstraunthaler, 2003; Shanbhag et al., 2020; Guiotto et al., 2020). A study showed that MSCs cultured in hPL supplemented media exhibit significantly higher proliferation rates and improved expansion kinetics compared with cells grown in media supplemented with FBS or HS (Bieback et al., 2009). Notably, another study found that 5% hPL stimulated MSCs proliferation by 2-fold than that in 2% hPL after 96 h incubation, suggesting a dose-dependence for hPL (Du et al., 2022).

The use of hPL hold high potential for tissue engineering and organoids cultivation. Nevertheless, reproducibility and regulatory safety continue to pose challenges in hPL usage. As Table 4 shows, the concentration of IGF and cortisol was more than two-fold higher in hPL (125 μg/L, 72.8–77.3 μg/L) than in FBS (40–72 μg/L, 20 μg/L), which may impact the efficacy of cell-based therapies if hormonal regulation is present (Peters et al., 2022; Miell et al., 1997). There is also a risk of transmitting infectious diseases (e.g., HIV, hepatitis) in both HS and hPL from human donors to cell lines (Schallmoser et al., 2020). Additionally, hPL remains subject to batch-to-batch variability and the presence of undefined animal-derived components. To prevent coagulation, hPL is typically supplemented with bovine or porcine-derived heparin (Weber et al., 2025). Nevertheless, variations among donors and risks of transmitting human disease introduces safety challenges (Wijerathna-Yapa et al., 2025).

### 2.3 Plant-based alternatives

Plant extracts and derivatives (e.g., soy protein, aloe vera, alfalfa) emerged with popularity in cell culture for their potential benefits, including promoting cell growth, supporting differentiation, and enhancing antioxidant and wound-healing properties (Georgiev et al., 2018; Pazos et al., 2004). Soy protein hydrolysates, for instance, produced *via* enzymatic digestion, have been found to enhance IgG levels in human cell culture (Wijerathna-Yapa et al., 2025). *Aloe vera* extracts are rich sources of essential amino acids, polysaccharides and glycoproteins, which can be used as an FBS alternative in cell culture media to promote mammalian cell proliferation and viability (Manirakiza et al., 2021). Besides, vitamins, catalase and superoxide contained in *aloe vera* can help protect cells from oxidative stress in *in vitro* culture (Tong et al., 2021; du Plessis and Hamman, 2014). *Aloe vera* extracts also contribute to tissue regeneration (Shalabi et al., 2015; Tong et al., 2021; du Plessis and Hamman, 2014; Manirakiza et al., 2021). A recent study showed that *aloe vera* extracts significantly promote MSCs differentiation to alleviate liver tissues damage (Farid et al., 2022). These results highlight its potential applications in regenerative medicine and wound healing studies (Farid et al., 2022). Notwithstanding, its use is limited by dose-dependent cytotoxicity and compatibility issues (Manirakiza et al., 2021; Badakhsh et al., 2014). *Aloe vera* at a lower concentration of 2.5% or 10% has been shown to improve the proliferation rates of human dermal fibroblasts, and MSCs, whereas anthraquinone derivatives found in *aloe vera* extracts induce apoptosis in human hepatocellular carcinoma (HepG2) (12.5 mg/mL) by mediating mitochondrial activity and P53 pathway (Farid et al., 2022).

Plant-derived supplements, however, present several critical concerns in mammalian cell culture that can affect experimental reproducibility and regulatory compliance (Manirakiza et al., 2021; Badakhsh et al., 2014). One major limitation is batch-to-batch variability, as the biochemical composition of plant extracts fluctuates due to differences in species, cultivation conditions, and harvest timing (Badakhsh et al., 2014). A comparative metabolomic study of 9 commercial hydrolysate products (4 plant- and 5 yeast-derived) revealed that only 15 out of 90 metabolites were common across all lots (Combe et al., 2024). The use of plant-derived supplements in clinical or biopharmaceutical contexts is hindered by regulatory hurdles, since undefined compositions and potential contaminants make it difficult to meet stringent quality and safety standards required for GMP applications (Fischer et al., 2012). Consequently, manufacturers prefer chemically defined media with rigorously controlled, well-characterized components rather than undefined botanical hydrolysates (Ho et al., 2021).

### 2.4 Algae-derived serum free media

*Galdieria sulphuraria*, a thermo-acidophilic red microalga, has shown promise as a source of protein-rich extracts in mammalian cell culture (Hütker et al., 2026; Eisenberg et al., 2025). Heat-treated protein extracts preserved proliferation and myogenic potential of murine C2C12 myoblasts under serum-free condition (Hütker et al., 2026). These extracts also enhanced growth of Chinese hamster ovary (CHO) cells, with performance approaching that of 5%–10% FBS (Eisenberg et al., 2025). These findings suggest that *G. sulphuraria*-derived proteins may serve as sustainable and scalable substitutes for animal-derived serum, although further optimization of the extract composition is necessary to fully match FBS in diverse cell culture applications (Eisenberg et al., 2025).

### 2.5 Serum free media

Serum free media (SFM) is classified into six types based on the absence of components, i.e., chemically defined (CD), chemically defined recombinant (CDR), chemically defined purified (CDP), protein-free (PF), animal component-free (ACF) and xeno-free (XF) media (Butler, 2015; Weber et al., 2025).

CD media, containing known, non-animal -derived components (Gstraunthaler, 2003), have been developed to support long-term culture of many established cell lines (e.g., HeLa cells, adipose-derived MSCs), though some sensitive cells may require adaptation (Bjare, 1992; Butler, 2015; Nessar et al., 2025). Adaptation to CD media often requires gradual weaning and multiple passages (Jang et al., 2022; Even et al., 2006). For example, the HEK293 cell line demands approximately 1 month to adapt, accompanied by altered growth kinetics and metabolic profiles (Jang et al., 2022). Similarly, CHO-K1 cells exhibit reduced viability during trypsinization, centrifugation, or subculturing, indicating the absence of protective factors typically provided by serum (Even et al., 2006). CDR media contain defined components originally derived from plants, animals or humans and produced as recombinant proteins (Weber et al., 2025). OptiVERO medium, supplemented with recombinant human transferrin and albumin, has been specifically optimized for VERO cell expansion and virus production (Alfano et al., 2020). A recent study validated that supplementing CDR media (below $ 1 per litre) with whey protein-derived components can support cell proliferation comparable to FBS formulations, suggesting a potential long-term paradigm shift in cell culture methodology (Mordor Intelligence, 2025). Protein extracts derived from red microalgae facilitate myoblasts (Hütker et al., 2026). CDP media are formulations in which all components and their concentrations are explicitly known and controlled, with no serum or hydrolysates (Weber et al., 2025). CD, CDR and CDP media formulations eliminate undefined human and animal-derived components, minimizing contamination risk and align with ethical requirements for animal-free culture in stem cell research and biopharmaceutical production (Lee D. Y. et al., 2022; Dai et al., 2024).

PF media, by definition, lack intact proteins or protein fractions derived from human and animal source, although they may not be fully chemically defined (Butler, 2015; Weber et al., 2025). PF media have been developed to cultivate various microorganisms in food safety research using minimal salt bases, but they often contain complex and undefined components (e.g., hydrolysates from plants or yeast) (Mosser et al., 2013). For instance, Plant Protein Hydrolysate (Aladdin Scientific) has been shown to support proliferation of CHO-320 cells, demonstrating that plant-derived hydrolysates serve as effective supplements in serum-free culture systems (Ballez et al., 2004). Additionally, PF media are used to culture hybridoma cells to enhance the homogeneity and purity of paired anti-human insulin mAbs for diabetes therapy, avoiding the extensive purification required when using FBS-containing media, where exogenous protein content can exceed mAbs by five-to-several-thousand-fold (Even et al., 2006; Kovár and Franĕk, 1984). Another study further reported that the addition of yeast hydrolysates and peptones increased CHO cell density by 70% and IgG production by 180% (Mosser et al., 2013). Notwithstanding, PF media contain no albumin or other protein, which could reduce contamination risk but often result in slower growth and lower viability (Bjare, 1992; Butler, 2015).

ACF media containing proteins derived from plant or microbial sources provide an ethical alternative while supporting growth rates comparable to those of serum-containing media for many cell types (Gstraunthaler, 2003). For example, Ex-Cell CHO Fusion medium (Sigma/Aldrich) omits animal-derived proteins for CHO-K1 culture and transfection analysis (Yamano-Adachi et al., 2020). Recombinant insulin, transferrin and selenium have been used in place of FBS for human MSCs culture, showing comparable proliferation and differentiation performance (Kolkmann et al., 2022). ACF media can support up to 130 billion cells per litre at a cost of $0.63 per litre, offering a major ethical and significant cost-effective advantage (Pasitka et al., 2024). XF media consist of animal-derived components only when they originate from the same species as the cultured cells, thereby reducing the risk of pathogen transmission (Weber et al., 2025). XF media are especially preferred in clinical cell therapy and regenerative medicine, where minimizing immunogenicity and meeting regulatory requirements are essential (McGrath et al., 2019). Compared with ACF media, XF media, however, often require more extensive optimization and higher costs to achieve similar cell growth performance (Hua et al., 2022).

In clinical and biopharmaceutical manufacturing, SFM is favoured for its precise composition, safety and reproducibility (Brunner et al., 2010; Newsom et al., 2025). Synthetic and defined formulations are easily optimized for specific cell lines or primary cells, but this strength also limits their broad applicability (Liu et al., 2023; Brunner et al., 2010). For instance, stemPro-34 SFM is highly specific for human hematopoietic cells derived from bone marrow or cord blood sources, whereas most differentiated specialized cells (e.g., MSCs, fibroblasts, or HEK293 cells) cannot be cultured (Butler, 2015). Since FBS is an exceptionally complex and nutrient-rich mixture, many of which are still not fully characterized or quantifiable in SFM formulations (Bjare, 1992). Investigating non-serum sources of growth factors, such as bioactive peptides and trace elements, is crucial for achieving greater consistency and scalability in SFM formulations (Wijerathna-Yapa et al., 2025).

## 3 Future perspectives of FBS alternatives and applications

Over recent decades, substantial progress has been made in the development of FBS alternatives to address the growing demand for reliability, health safety, ethical and environmental responsibility in mammalian cell culture (Witzeneder et al., 2013; Golshan et al., 2025; Wijerathna-Yapa et al., 2025; Gstraunthaler, 2003) (Table 3). Oredsson universal replacement (OUR) medium, for instance, is an open-access, XF cell culture medium formulated to support the cultivation of a wide range of human normal cells including VERO, A549, Jurkat, HeLa, and cancer-associated fibroblasts in both 2D and 3D systems (Weber et al., 2024; Rafnsdóttir et al., 2023; Malakpour-Permlid et al., 2025). Emerging suppliers are focusing on enhancing quality and specialization by adding or removing specific components from FBS products (Table 6). In the future, optimized, application-specific FBS substitutes will be required to meet the biological, metabolic, and regulatory demands of modern mammalian cell culture (Subbiahanadar Chelladurai et al., 2021). Drawing on insights from prior studies on FBS alternatives, this review proposes the main development directions, capacity-building strategies and incentive measures to facilitate the transition toward FBS alternatives.

### 3.1 Tailoring FBS alternatives for specific applications

**FIGURE 2:**
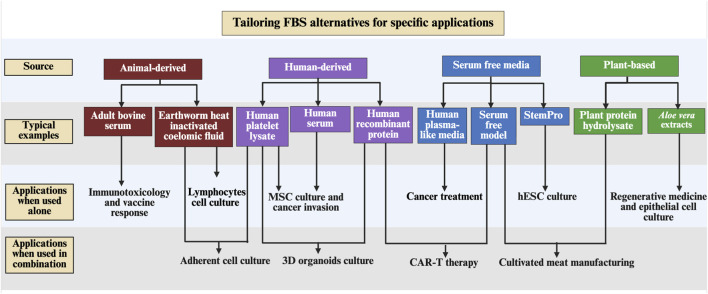
Tailoring FBS alternatives for specific applications. Adult bovine serum is preferred in immunotoxicology and vaccine response studies, while earthworm heat inactivated coelomic fluid combined with human platelet lysate supports adherent cell cultures. Human serum is particularly effective for MSC culture and cancer cell invasion assays, and *Aloe vera* extracts are used in regenerative medicine and epithelial cell culture. Serum free model supplemented with plant-derived protein hydrolysates has been applied in cultivated meat production. Besides, serum free media with human recombinant proteins is critical for enhancing CAR-T therapy performance. Human plasma-like media are increasingly favoured in 3D organoids culture and cell-based therapies for reducing immunogenicity, xenogeneic antigens, and pathogen risks. Created in BioRender (https://biorender.com/). CAR-T, Chimeric antigen receptor T; hESC, human embryonic stem cells; MSC, mesenchymal stromal cells.

OUR medium has been successfully applied to 27 human and animal cell lines and is considered one of the most promising alternatives to FBS (Oredsson et al., 2025). In practice, laboratories typically select a base medium suited to most of their target cells and then customize supplements to meet the specific requirement of cells (Cantor et al., 2017; Wijerathna-Yapa et al., 2025). For instance, human-derived supplements and scaffold materials have been used to maintain viability and invasiveness in 3D tumour and stem cell spheroids, demonstrating the importance of tailored culture conditions for complex tissue-like architectures (Monteiro et al., 2020). In parallel, when the species origin of an FBS substitute matches that of the cultured cells, superior proliferation performances have been observed compared with commercial FBS at equivalent concentrations (Lee et al., 2023). These findings highlight the critical need to develop customized FBS alternatives that closely mimic the compositions of commercial FBS by supplementing or removing specific components. Such approaches are essential to ensure reliable, safe, and ethically responsible mammalian cell culture across diverse applications, including 3D organoids culture, disease diagnostics, cell-based therapies and cultured meat production (Figure 2; Table 5).

**TABLE 5:** Advances in tailoring culture media for 3D organoids culture, disease diagnostics, cell-based therapy and cultivated meat production.

Application area	Tailoring media	Benefit(s)	References(s)
3D organoids culture	hPL supplemented with recombinant laminin	Retinal organoids culture	Rashidi et al. (2022)
Disease diagnostics	hPL	Patient-derived cancer chemosensitivity assay	Du et al. (2022)
	AIM-V, X-VIVO^TM^	Immunological diagnostics	Cochrane et al. (2024)
	Specialized FBS (Exosome-depleted), SFM	EV-based biomarker assay	Urzì et al. (2022)
Cell-based therapy	hPL with GMP-grade protocols	Clinical-grade MSC expansion	Czapla et al. (2019), Golshan et al. (2025)
	HPLM	Improved drug sensitivity in cancer cell model	Ackermann and Tardito (2019), Torres-Quesada et al. (2022)
	Essential 8™ (E8) media	Maintaining differentiation potential of hiPSCs	Chen et al. (2011)
	mTeSR1 and StemPro SFM	hESCs culture	Akopian et al. (2010)
	SFM with human cytokines and recombinant proteins	CAR-T cell therapy	Chen et al. (2024), Wijerathna-Yapa et al. (2025)
Cultivated meat	Food-grade SFM with whey protein hydrolysates	Bovine myoblast proliferation	Segawa et al. (2025)
	Recombinant albumin produced in *Pichia pastoris*	Providing scalable carrier protein for myotube formation	Kim et al. (2025)
	Formulated SFM	Supporting myogenic differentiation	Stout et al. (2022)

AIM-V, Advanced Iscove’s Modified Dulbecco’s Medium-V; CAR-T, Chimeric antigen receptor T; EV, extracellular vesicle; hESC, human embryonic stem cells; HPLM, human plasma-like media; hiPSC, human induced pluripotent stem cells; hPL, human platelet lysate; GMP, Good Manufacturing Practice; MSC, mesenchymal stromal cells; SFM, serum free media.

#### 3.1.1 3D organoid cultures

3D organoids are derived from organs, tumours, or stem cells that recapitulate native tissue structure and function for studying disease development and personalized medicine (Figure 2; Table 5) (Takebe and Wells, 2019; Górska et al., 2024; Wu et al., 2021). Their reliability in research depends on minimizing heterogeneity including any introduced during culturing, and where altered FBS composition can reduce reproducibility (Wu et al., 2021). Researchers generated retinal organoids using hPL and human recombinant laminin in place of FBS (Even et al., 2006; Wu et al., 2021). SFM increasingly replaces FBS to maintain the tumour microenvironment in 3D cell models (Schnitzler et al., 2015; Czapla et al., 2019; Golshan et al., 2025). 3D primary human hepatocytes (PHHs) cultured in an ACF media with normoglycemic formulations (5.5 mM glucose and 0.58 ng/mL insulin) exhibited viability and cytochrome P450 functional performance comparable to cells cultured in FBS-containing media (Mickols et al., 2025). A serum free model supplemented with soluble Wnt mimetics, carriers, and small-molecule modulators has been shown to improve the productivity and stability of Wnt3A, a critical growth factor for organoid expansion (Rashidi et al., 2022). These advances highlight a substantial step forward in establishing more reproducible and ethically sound 3D organoid research.

#### 3.1.2 Human disease diagnostics

Recent progress in tailoring FBS alternatives has improved human diagnostic performance (Figure 2; Table 5) (Rashidi et al., 2022; Czapla et al., 2019; Golshan et al., 2025; Ackermann and Tardito, 2019; Torres-Quesada et al., 2022). For example, hPL supports the expansion and chemosensitivity profiling of primary cancer cells by eliminating xenogeneic bovine components that can interfere with diagnostic readouts (Du et al., 2022). SFM, including Advanced Iscove’s Modified Dulbecco’s medium-V (AIM-V) and X-VIVO^TM^ SFM, has been widely adopted in immunological diagnostics (Cochrane et al., 2024). In extracellular-vesicle (EV)-based biomarker discovery, exosome-depleted FBS or SFM is essential for improving the specificity of EV-mediated assays (Urzì et al., 2022). Collectively, these approaches demonstrated the importance of aligning media formulations with diagnostic applications to reduce artefacts and achieve clinical reproducibility, and they warrant further study (Du et al., 2022; Cochrane et al., 2024; Urzì et al., 2022).

#### 3.1.3 Cell-based therapy

Trends in FBS alternatives have also focused on creating more physiologically relevant conditions to optimize cell function and improve therapeutic outcomes (Figure 2; Table 5) (Weber et al., 2025; Golshan et al., 2025). The metabolic and functional demands of human cells can differ markedly from those of animal cells: for example, human plasma-like media (HPLM) that comprises 60 metabolites (e.g., amino acids, lipids) at physiologically relevant concentrations, demonstrated improved support for human cell culture (Ackermann and Tardito, 2019; Torres-Quesada et al., 2022). This leads to more accurate assessments of drug efficacy and cellular bioenergetics, especially in cancer models, and can reveal drug effects not seen in traditional media (Ackermann and Tardito, 2019; Torres-Quesada et al., 2022). In addition, chemically synthetic and XF media, i.e., Essential 8 ^TM^ (E8, Gibco) media provide a fully defined environment to promote derivation efficiency of human induced pluripotent stem cells (hiPSCs) for long-term culture (Chen et al., 2011). Human embryonic stem cells (hESCs) have been successfully adapted to SFM mTeSR1 (STEMCELL Technologies) and StemPro (Gibco), with morphology and differentiation capacity maintained (Akopian et al., 2010). SFM optimized for suspension HEK293 cell cultures increased Adeno-associated virus (AAV) yield and purity for clinical-grade manufacture by removing serum proteins (Grieger et al., 2016). SFM supplemented with human cytokines (e.g., IL-2) and recombinant proteins can enhance the proliferation and genetic modification efficiency of chimeric antigen receptor T (CAR-T) cells by decreasing exhaustion markers (Wijerathna-Yapa et al., 2025).

#### 3.1.4 Cultivated meat manufacturing

Given the concerns about traditional meat production, there is considerable interest in developing proliferation and differentiation of stem cells *in vitro* to produce edible meat, also known as cultivated meat (Messmer et al., 2022). Producing cultured meat relies on mammalian cells that require carefully controlled environments and complex growth media containing amino acids, vitamins, lipids and growth factors (Lee et al., 2023) (Figure 2; Table 5). In addition, unlike plants, animal-cells cannot self-assemble into structured tissues; achieving a meat-like texture requires scaffolds to support cell adhesion, alignment, and differentiation into muscle and fat tissues (Lee et al., 2024). Hence, the development of FBS alternatives for cultivated meat production has focused on providing essential nutrients and growth factors for muscle and fat cell proliferation while permitting food-grade safety (Segawa et al., 2025; Kim et al., 2025; Takebe and Wells, 2019; Górska et al., 2024; Wu et al., 2021). Studies have shown that formulated differentiation SFM supports the myogenic differentiation of 3D muscle organoids without transgene expression, demonstrating its effectiveness in producing bioartificial muscle constructs for cultured beef (Stout et al., 2022). Plant- and food-derived protein hydrolysates (e.g., whey protein) have been employed to enhance bovine myoblasts under serum-free conditions (Segawa et al., 2025). Recombinant carrier proteins, such as albumin produced in *Pichia pastoris*, have provided scalable and non-animal-derived alternatives that maintain myotube formation (Kim et al., 2025; Dai et al., 2024; Stout et al., 2022).

### 3.2 Improving the quality and specialized formulation of FBS

Significant efforts have been made to improve FBS quality by reducing contaminants (endotoxin, viruses, mycoplasma), minimizing undefined components, and developing specialized formulations to standardize FBS performance (Even et al., 2006; Wu et al., 2021). Table 6 presents the origin, supplier, special features, and price of the premium-quality, specialized commercial FBS products. For example, Appleton Woods offers FBS with endotoxin levels of <1 Endotoxin Units (EU)/mL ($597/500 mL), eliminating bacterial lipopolysaccharide contamination in sensitive cell culture. Treatment of FBS with charcoal removes steroid hormones, supplied by Gibco ($176/500 mL), making it suitable for hormone-sensitive cancer studies. Hyclone has developed FBS products with low lot-to-lot variation (e.g., ±10%) and has tested for IgG reduction to ensure consistent performance ($886/500 mL). Premium FBS products may undergo additional filtration and gamma irradiation for contaminant removal (e.g., mycoplasma and adventitious agents), as well as provide traceability of their origin and collection methods for quality control. Nevertheless, their compositions remain fundamentally undefined, and batch variability persists, which can affect cell culture reproducibility (Pecora et al., 2020).

**TABLE 6:** Origin, supplier, price and features of the premium-quality, specialized FBS products.

Product	Origin	Supplier	$ per 500 mL	Special features
FBS EU compliant	South America	Appleton Woods	597	Endotoxin-reduced
FBS Charcoal Stripped	U.S.A.	Gibco	176	Charcoal-stripped
Characterized FBS	U.S.A.	Hyclone	886	Low lot variability
Premium FBS	South America	Corning	671	Exosome-depleted

EU, European Union; FBS, foetal bovine serum.

### 3.3 Optimization of current FBS alternatives

If the use of FBS could be replaced—at least in academic research—for culturing mammalian immortalized cell lines and primary cells, the overall demand for FBS could be reduced substantially (Wijerathna-Yapa et al., 2025; Subbiahanadar Chelladurai et al., 2021). Animal-derived sera such as ABS and horse serum (HSr) have continued to be explored as partial or context-specific alternatives to FBS due to their compositional similarity (Yu et al., 2013). Because it is derived from the same species as FBS, ABS most closely mimics the complex composition of FBS among animal-derived sera, helping mammalian cells, i.e., MSC, maintain more physiologically relevant behaviour and experience less adaptation stress when switching from FBS (Shehzadi et al., 2024). In the future, efforts should focus on optimizing ABS performance through implementing functional batch testing to mitigate variability, and combining ABS with recombinant growth factors or serum-free basal media (Shehzadi et al., 2024). Heat inactivation is a controversial step because it can reduce heat-labile growth factors, yet it is a recommended optimization step for complement-sensitive assays (Giard, 1987).

Improving standardization to reduce donor- and batch-to-batch variability is a key direction in the development of human-derived alternatives (Immalaraju et al., 2025). For instance, hPL should ideally be prepared from a limited number of pooled platelet units to minimize the risk of donor-to-donor disease, and donors should be excluded if relevant exposure is identified in their medical history (Guiotto et al., 2020). Moreover, combining supplements from different sources may be a viable strategy. The lack of attachment factors in HI-CF can be compensated by the addition of hPL (Subbiahanadar Chelladurai et al., 2021). A thorough test of the xenogeneic components of hPL-HI-CF is needed before applying the combination into translational research, pharmacological studies, or industrial production (Tancharoen et al., 2019). Alternatively, combining HI-CF with cell growth and attachment factors in SFM (f-HI-CF) has been shown to effectively support the proliferation of various adherent cell lines, e.g., A549 cell line, HeLa (adenocarcinoma-derived epithelial cells) and C2C12 (Rossan Mathews et al., 2024). The Biotechnology Industry Research Assistance Council (BIRAC) in India highlighted its commercial potential of a precise formulation (HI-CF (1%), selenium (0.0633 µM), fetuin (0.123 mM), insulin (1.726 µM), and transferrin (15.177 µM)) as a potential alternative to FBS in animal cell culture (BIRAC, 2025).

Plant-derived protein hydrolysates are increasingly used as serum-free supplements due to ethical considerations (the 3Rs: Replacement, Refinement, and Reduction), reduced animal-origin contamination, and improved sustainability. Protein hydrolysates from sources such as corn, potato, soy, and algae support cell proliferation under serum-free conditions; for example, UltraPep® Soy shows excellent filterability while supporting CHO-K1 growth (Babcock et al., 2010). 10% Prolifix enables adaptation of epithelial cell lines (LLC-PK1, Caco-2) to SFM (Pazos et al., 2004). Recently, plant-derived soy hydrolysates or gelatine have been incorporated into XF culture systems as surface coatings to support cell adhesion and maintain long-term (90 days) culture of human and murine fibroblasts (Mogilever et al., 2025). Additionally, plant-derived FGF2 and human EGF efficiently directed the differentiation of human-induced pluripotent stem cells (hiPSC) differentiation into neural stem cells, which further differentiated into neuronal and glial lineages, demonstrating the feasibility of animal-free culture systems for stem cell applications (Lee Y. et al., 2022). Overall, despite challenges in defining optimal dosages, these developments demonstrate the potential of plant-derived supplements to replace FBS and underscore the need for cell-type specific optimization.

Recent innovations in SFM have improved ethical compliance, GMP compatibility, and positioned these systems as key future directions in mammalian cell culture (Even et al., 2006). Notably, SFM has proven effective for culturing parental cells, such as splenocytes and myelomas, immediately following cell fusion during hybridoma production (Kovár and Franĕk, 1984). However, identifying sources of growth-supporting components, such as bioactive peptides and trace elements, remains critical for improving the consistency and scalability of SFM (Brunner et al., 2010). A study of primary bovine myoblasts showed that only 2 of 7 tested SFMs supported proliferation comparable to the FBS control (Kolkmann et al., 2020). To address this limitation, human-origin hydrolysates and chemically defined protein/lipid supplements, as well as adaptive culture that gradually transition cells from low-serum to serum free conditions have been developed to retain broad growth support while improving consistency and ethical acceptability (Rafnsdóttir et al., 2023).

Hence, future directions for developing FBS alternatives should focus on minimizing batch-to-batch variability, optimizing formulations for specific applications, and advancing fully defined and scalable media to enable reliable, ethical, and clinically relevant mammalian cell culture (Subbiahanadar Chelladurai et al., 2021).

### 3.4 Database construction, incentive mechanisms establishment, and selection guidance for FBS alternatives

#### 3.4.1 Database and platform construction and maintenance

To facilitate the interactive exchange of information and expertise on FBS alternatives, several serum-free or defined culture media (inter)national databases for researchers were created, such as https://fcs-free.org/fcs-database (3Rs Centre Utrecht, 2024). However, the FCS-free database acknowledges that information for some commercial products and suppliers may be incomplete and that not all data are fully verified, indicating potential inconsistencies in data coverage across database entries (Disclaimer, 2024). This highlights the urgent need to establish a centrally managed and rigorously curated database with standardized metadata and comprehensive coverage of FBS alternatives, in order to enhance communication of emerging replacement strategies and systematically track progress within the field (Rosolowski et al., 2025). Support and participation from various stakeholders, including government, animal welfare organizations, academic communities, and commercial company is essential. For instance, SOPs of established chemically defined SFM should be made readily available through publications and databases.

#### 3.4.2 Innovative technologies and interdisciplinary advancement

Optimising cell culture media is a crucial yet complex task, owing to the nonlinear interactions among the different components in mammalian cell growth (Wijerathna-Yapa et al., 2025). Studies have shown that machine learning models, such as artificial neural networks build quantitative relationships between media compositions and critical quality attributes in cell metabolism (Kavoni et al., 2025; Wijerathna-Yapa et al., 2025). It is suggested that machine learning and synthetic biology methods should be applied to optimize media composition and to produce monoclonal antibodies with higher yield and improved quality (O’Flaherty et al., 2020; Kavoni et al., 2024). Other innovative and applicable technologies include omics-based profiling, high-throughput screening, 3D bioprinting and organ-on-a-chip systems, *etc.* (Combe et al., 2024; Baltazar et al., 2023; Weener et al., 2024; Ali et al., 2024).

#### 3.4.3 Incentive mechanisms for the adaptation of FBS alternatives

The reduction and replacement of FBS use with alternatives are becoming feasible as hPL and other serum-free substitutes have demonstrated performance comparable to that of FBS, supporting MSCs proliferation without compromising phenotypic stability, and enhancing monoclonal antibodies production in hybridoma cell lines (Even et al., 2006; Guiotto et al., 2020). However, the adoption of these alternatives in clinical practice, therapy, and broader scientific research remains limited by insufficient incentives to depart away from established FBS protocols (Hope and Bailey, 2025). Academic communities and administrative government should therefore collaborate to establish clear regulatory guidance, dedicated funding, and appropriate incentives to promote the adaptation of ethically and scientifically superior FBS alternatives (Gstraunthaler et al., 2013). Recent efforts have focused on developing SFM to encourage the scientific community to adopt efficient and ethical practices for cultivating diverse mammalian cell lines (Table 7). As previously noted, OUR medium has been widely used in culturing human and animal cell lines (Oredsson et al., 2025). A CD medium supplemented with TrypLE has been optimized to support HeLa cell growth (Nessar et al., 2025). Besides, recombinant human albumin has been incorporated into ACF media to culture multiple human and murine adherent cell lines (Mogilever et al., 2025). Another CD formulation, LM7, enhances the proliferation of immortalized lamb muscle cells (ILMSs) (Amirvaresi and Ovissipour, 2025). Furthermore, Surge SFM has been developed to support large-scale expansion of human primary keratinocyte (HPF) and skin substitute production (Ghio et al., 2023).

**TABLE 7:** Examples of recently developed SFM for the culture of diverse cell line.

Media	Cell lines	References
OUR medium	27 cell lines including human CaCo-2, JIMT-1, HeLa, MCF-7, A549, Jurkat, mouse L929, rat C6, *etc.*	Oredsson et al. (2025)
CD media with recombinant TrypLE	HeLa	Nessar et al. (2025)
LM7	ILMCs	Amirvaresi and Ovissipour (2025)
ACF media with recombinant human albumin	Murine NIH 3 T3, murine MC 3 T3 E1, murine C2C12 and HFF	Mogilever et al. (2025)
Surge SFM	HPF	Ghio et al. (2023)

ACF, animal component-free; CD, chemically defined; HFF, human foreskin fibroblasts; HPF, human primary keratinocyte; ILMCs, immortalized lamb muscle cells; OUR, Oredsson universal replacement; SFM, serum free media.

#### 3.4.4 Guidance for FBS alternatives selection

**FIGURE 3:**
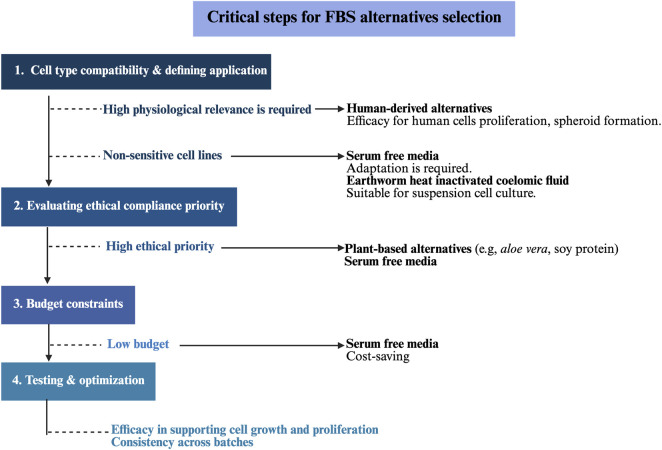
Critical steps for selecting FBS alternatives. Selecting a suitable alternative to FBS requires considering cell type, regulatory needs, and research application and budget limitation. The process begins by checking compatibility with specific cell type and identifying application. For example, human-derived alternatives are better for enhanced proliferation performance and cancer invasion study in human cells. But pathogen transmission testing is needed. Secondly, plant-derived alternatives and serum free media are more suitable if ethical consideration is a priority. Serum free media is a more cost-saving option. Finally, compatibility testing and optimization are required to verify that the chosen alternative effectively supports cell growth and yields consistent results across batches. Created in BioRender (https://biorender.com/).

Selecting an appropriate alternative to FBS depends on several factors including cell type, research application, regulatory needs, ethical considerations, and budget constraints (van der Valk, 2022; Lee et al., 2023). Considering the above progress and experiences in FBS alternatives development, we proposed a protocol for FBS selection (Figure 3). The first step is to define the specific cell type and clinical application. For instance, using human-derived alternatives (e.g., HS, hPL) may improve physiological relevance and enhance proliferation performance in human fibroblasts and MSCs culture (Peters et al., 2022; van der Valk, 2022). Besides, HS is more effective for spheroid formation and cancer invasion study (Heger et al., 2018). However, HS and hPL pose a risk of pathogen transmission (e.g., hepatitis) and therefore require rigorous testing for regulatory compliance (Astori et al., 2016). SFM is versatile but may require adaptation for primary cell lines (Butler, 2015). For example, PHH spheroids were gradually adapted to ACF media over 3 weeks to achieve comparable performance to that of spheroids cultured under FBS-containing conditions, starting with a medium composed of 50% serum-free formulation (Mickols et al., 2025). HI-CF may support suspension cells but has limitations for adherent cells due to the presence of fibrinolytic enzymes (Rossan Mathews et al., 2024). Secondly, if ethical compliance is a priority consideration in your project, plant-based alternatives (e.g., aloe vera, soy protein) and SFM are more closely aligned with 3R standards (Farid et al., 2022). Budget constraint is another critical consideration. SFM for routine cell culture are generally more cost-effective (i.e., Invitrogen $197/500 mL, OUR medium $113/500 mL) than FBS-containing media (Brunner et al., 2010; Oredsson et al., 2025). Notably, SFM designed for cell therapy or requiring GMP compliance represent an exception, exemplified by HIMEDIA STEMin1 Defined SFM ($1027/500 mL) for human MSCs expansion, in which high-purity recombinant proteins, cytokines and growth factors (e.g., FGF-2 and TGF- β) account for the bulk of the cost (Quek et al., 2024). By contrast, GMP-grade FBS undergoes extensive testing for sterility, endotoxins and viral contaminant, whereas unavoidable batch-to-batch variability can still affect reproducibility, thereby increase overall production costs (van der Valk et al., 2018). Lastly, testing and optimization are needed to ensure that the selected alternative is effective in supporting cell culture and yields consistent results across batches. In summary, assessing factors, such as cell types, ethical compliances, growth requirements, compatibility, regulatory safety, and cost, with specific demands, followed by testing and optimizing protocols, are needed for optimal selection.

## 4 Conclusion

FBS has historically been a cornerstone of cell culture due to its rich supply of growth factors and nutrients; however, ethical concerns, batch variability, biosafety risks, environmental impact, and rising costs have intensified the demand for reliable alternatives. Since the late 20th century, diverse FBS substitutes, including animal- and human-derived supplements, plant-based hydrolysates, and serum-free or chemically defined media, have emerged, each offering distinct advantages and limitations depending on the target cell types and applications. Increasing evidence indicates that species-matched supplements and application-specific formulations can enhance cell proliferation and functional performance while minimizing xenogeneic and regulatory risks. Although specialized FBS formulations remain valuable for certain niche applications, the overall trend favours GMP-compliant, XF, and ethically sourced media to improve reproducibility and translational relevance. Accordingly, future development of FBS alternatives should prioritize batch consistency, scalability, defined composition, and application-specific optimization. In parallel, the establishment of shared databases and communication platforms, integration of innovative technologies, implementation of incentive mechanisms, and standardized selection protocols will be essential to accelerate the dissemination and adoption of FBS alternatives across research, biomanufacturing, and clinical settings.
